# Systemic Metabolomic Remodeling in Pressure Overload-Induced Heart Failure Indicates Modulation of a Gut–Liver–Heart Axis by the Adiponectin Receptor Agonist ALY688

**DOI:** 10.3390/metabo16010038

**Published:** 2026-01-01

**Authors:** Yubin Lei, Benjie Li, Tori Gosse, Sungji Cho, Hye Kyoung Sung, Jiarui Chen, Gary Sweeney

**Affiliations:** 1 Department of Biology, York University, Toronto, ON M3J 1P3, Canada; 2 Guangdong Provincial Key Laboratory of Food, Nutrition and Health, Department of Nutrition, School of Public Health, Sun Yat-sen University, Guangzhou 510080, China

**Keywords:** adiponectin, heart failure, multi-tissue metabolomic, gut-derived metabolites, gut–liver–heart axis, therapeutic

## Abstract

Background/Objectives: Numerous studies have documented cardioprotective effects of adiponectin in animal models of cardiometabolic disease (CMD). Adiponectin receptor agonist ALY688 has demonstrated functional significance against pressure overload-induced cardiac remodeling events in a mouse model of heart failure with reduced ejection fraction (HFrEF), potentially through modulation of the systemic metabolome. However, the specific metabolites and their pathophysiological contribution to cardioprotection in cardiac hypertrophy or heart failure remain unclear. This study aimed to characterize systemic metabolic alterations across five tissues in HFrEF and determine how ALY688 modifies these pathways to mediate cardioprotection in the transverse aortic constriction (TAC) model. Methods: Targeted metabolic profiling was performed on heart, liver, muscle, epididymal white adipose tissue (eWAT), and serum collected five weeks post-surgery from wild-type male C57BL/6 mice. Mice underwent either Sham or TAC-induced left ventricular pressure overload, with or without daily subcutaneous ALY688 administration. Metabolites were quantified using liquid chromatography–tandem mass spectrometry (LC–MS/MS) and statistically analyzed at the tissue level. Results: Consistent with pathological cardiac remodeling, the comprehensive metabolomic analysis revealed that TAC induced widespread disruption of systemic metabolic homeostasis. ALY688 treatment significantly modified several key metabolite classes, including triglycerides (TGs) and glycosylceramides (HexCer). Notably, ALY688 also altered multiple gut-derived metabolites, including trimethylamine N-oxide (TMAO), 5-aminovaleric acid (5-AVA), and glycodeoxycholic acid (GDCA), highlighting a potential gut–liver–heart axis mediating its cardioprotective effects. Conclusions: These findings demonstrate that ALY688 mitigates TAC-induced metabolic dysregulation across multiple tissues. The identified metabolic signatures suggest that ALY688 exerts cardioprotective effects, at least in part, through restoration of systemic metabolic homeostasis and engagement of a gut–liver–heart metabolic axis. These results provide mechanistic insight into adiponectin receptor agonism and support further exploration of ALY688 as a potential therapeutic strategy for HFrEF.

## 1. Introduction

Heart failure (HF) affects more than 55.5 million people and is a leading cause of morbidity and mortality, characterized not only by structural and functional decline of the myocardium but also by profound disturbances in energy metabolism [1,2,3,4]. Under conditions of pressure overload, the failing heart undergoes metabolic remodeling that impairs substrate flexibility, depletes triglyceride energy reserves, disrupts phospholipid membrane integrity, and promotes accumulation of lipotoxic intermediates such as ceramides and cholesterol esters [4,5,6]. These maladaptive changes extend beyond the heart, engaging skeletal muscle, liver, and epididymal white adipose tissue (eWAT) through circulation, ultimately contributing to systemic metabolic stress.

Although metabolic dysfunction is increasingly recognized as a driver of HF progression, therapeutic strategies that directly reprogram systemic metabolism remain limited [4,5,7]. Peptide-based approaches, such as the adiponectin receptor agonist ALY688, have emerged as a promising therapeutic agent, with evidence suggesting that the beneficial effects range from insulin sensitivity and lipid homeostasis to reduced pressure overload-induced fibrosis, hypertrophy, inflammation, and metabolic dysfunction [8,9,10,11]. However, the tissue-specific and systemic metabolic effects of ALY688 in the context of pressure overload-induced heart failure with reduced ejection fraction (HFrEF) remain poorly understood.

Metabolomic analysis using liquid chromatography coupled with tandem mass spectrometry (LC–MS/MS) has emerged as a powerful tool to investigate metabolic perturbations at the systemic level [11,12,13]. It enables the discovery of biomarkers, elucidation of molecular mechanisms underlying disease progression, and identification of therapeutic targets [14]. A growing body of multi-organ metabolomics research has highlighted the involvement of gut microbiota-derived metabolites, including bile acids, amino acid derivatives, and short-chain fatty acids, in systemic metabolic rewiring and cardiometabolic crosstalk, underscoring the pivotal roles of gut–heart and gut–liver axes in HF pathophysiology [15,16,17,18]. These insights highlight the need to better understand how therapeutic modulation of metabolic pathways, including through agents such as ALY688, may restore metabolic homeostasis at both the cardiac and systemic levels.

In the present study, we employed targeted metabolomic analysis using LC–MS/MS to quantitatively analyze the level of more than 600 metabolites across heart, liver, skeletal muscle, eWAT, and serum. This comprehensive multi-organ approach enabled systemic characterization of the metabolic alternations induced by pressure overload in HF. We aimed to identify tissue-specific and circulating metabolic signatures that are associated with cardiac dysfunction and systemic metabolic stress. Furthermore, we investigated whether ALY688 administration could normalize maladaptive metabolic patterns, restore substrate flexibility, and, thereby, alleviate cardiac hypertrophy, fibrosis, and inflammation.

## 2. Materials and Methods

### 2.1. Experimental Animals and Surgical Induction of Pressure Overload

All animal experiments were approved by the York University Animal Care Committee, and animal facilities conformed to the Canadian Council on Animal Care guidelines. Animal experiments were performed as previously described [11]. All mice used in this study were wild-type male C57BL/6 strain mice (10–12 weeks old at day 0) from Charles River Laboratories (Montreal, QC, Canada). The mice were allowed to acclimatize for a minimum of two weeks upon arrival. Experimental animals were housed in a temperature-controlled environment under 12 h light and 12 h dark conditions, with free access to food (a standard rodent chow diet, Lab Diet 5015) and water. All mice were then randomized into one of three experimental groups: (1) Sham group subjected to vehicle (sterile 0.9% saline) injection and Sham surgery; (2) transverse aortic banding (TAC) group subjected to vehicle injection and minimally invasive TAC surgery; and (3) TAC with ALY688 (TAC-A) group subjected to ALY688 3 mg/kg injection and TAC surgery. Daily vehicle or ALY688 treatment via subcutaneous injection began two days prior to surgery. Mice continued to receive daily subcutaneous injections for the duration of the study. All mice were euthanized five weeks post-surgery, and whole blood samples were collected in tubes without anticoagulant and kept at room temperature for one hour prior to centrifugation at 2000× *g* for 10 min for serum collection. The heart, liver, skeletal muscle, and eWAT tissue were rinsed in sterile PBS and snap frozen for further analysis.

### 2.2. Targeted Metabolomics Analysis by LC–MS/MS

Targeted metabolomic profiles were determined in the above-mentioned samples isolated five weeks post-surgery by The Metabolomics Innovation Centre (Edmonton, AB, Canada). Each tissue sample was accurately weighed, and the weighed mass was recorded, followed by homogenization with a 3-fold volume of tissue exaction buffer (extraction buffer was prepared by mixing 85 mL methanol and 15 mL of 10 mM phosphate buffer). After homogenization, samples were centrifuged at 14,000 rpm for 20 min, and the supernatants were transferred into new Eppendorf tubes for further LC–MS/MS sample preparation. We applied a targeted quantitative metabolomics approach to analyze the serum samples using a combination of direct injection mass spectrometry (MxP500 Kit) with a reverse-phase LC–MS/MS kit. The kit is a commercially available assay from BIOCRATES Life Sciences AG (Innsbruck, Austria). This kit, in combination with an ABI 5500 Q-Trap (SCIEX, Framingham, MA, USA) mass spectrometer, can be used for the targeted identification and quantification of up to 630 different endogenous metabolites, including amino acids, acylcarnitines, biogenic amines, bile acids, organic acids, steroids, diglycerides (DGs), triglycerides (TGs), phosphatidylcholines (PCs), lysophosphatidylcholines (LysoPCs), sphingomyelins (SMs), ceramides (Cers), and cholesteryl esters (CEs) and sugars. The method used combines the derivatization and extraction of analytes with selective mass-spectrometric detection using multiple reaction monitoring (MRM) pairs. Isotope-labeled internal standards and other internal standards are integrated in the kit plate filter for metabolite quantification. The MxP500 kit contains a 96 deep-well plate with a filter plate attached with sealing tape and the reagents and solvents used to prepare the plate assay. The first 14 wells in the kit were used for one blank, three zero samples, seven standards, and three quality control samples provided with each kit. All the serum samples were analyzed with the kit using the protocol described in the user manual. Briefly, serum samples were thawed on ice and were vortexed and centrifuged at 13,000× *g*. A total of 10 µL of each serum sample was loaded onto the center of the filter on the upper 96-well kit plate and dried in a stream of nitrogen. Subsequently, 20 µL of a 5% solution of phenyl-isothiocyanate was added for derivatization. After incubation, the filter spots were dried again using an evaporator. Extraction of the metabolites was then achieved by adding 300 µL methanol containing 5 mM ammonium acetate. The extracts were obtained by centrifugation into the lower 96 deep-well plate, followed by a dilution step with kit MS running solvent. Mass spectrometric analysis was performed on an API5500 Qtrap^®^ tandem mass spectrometry instrument (SCIEX, Framingham, MA, USA) equipped with a solvent delivery system. The samples were delivered to the mass spectrometer by an LC method, followed by a direct injection (DI) method. The Biocrates MetIQ software (Biocrates Life Sciences AG, Innsbruck, Austria; https://biocrates.com/metidq-software, accessed on 23 December 2025) was used to control the entire assay workflow from sample registration to automated calculation of metabolite concentrations to the export of data into other data analysis programs. A targeted profiling scheme was used to quantitatively screen for known small molecule metabolites using multiple reaction monitoring, neutral loss, and precursor ion scan.

### 2.3. Data Analysis and Statistics

Pairwise comparisons between TAC vs. Sham and TAC-A vs. TAC were performed for all 630 annotated metabolites across five tissues. Differential metabolites were defined as those with *p* < 0.05 and fold change > 1.2 or <0.83. For metabolites with missing or undetected concentration values, one-half of the limit of detection (LOD) was imputed to minimize bias while retaining biological relevance. To assess group separation and identify discriminative metabolic signatures, partial least squares discriminant analysis (PLS-DA) was applied as a supervised multivariate method. Multinomial logistic regression (MLR) was subsequently performed using normalized metabolite concentrations as continuous predictors to model three-category group classification as previously described [14]. Model-predicted probabilities were computed across a normalized concentration range and visualized as probability curves. Diagnostic thresholds were defined where the predicted probability of a class reached 50%, and class-dominant regions were highlighted where the probability of TAC-A exceeded the combined probability of the Sham and TAC groups.

## 3. Results

### 3.1. Pressue Overload Induces Tissue-Specific Metabolic Remodeling Across Tissues

**Figure 1:**
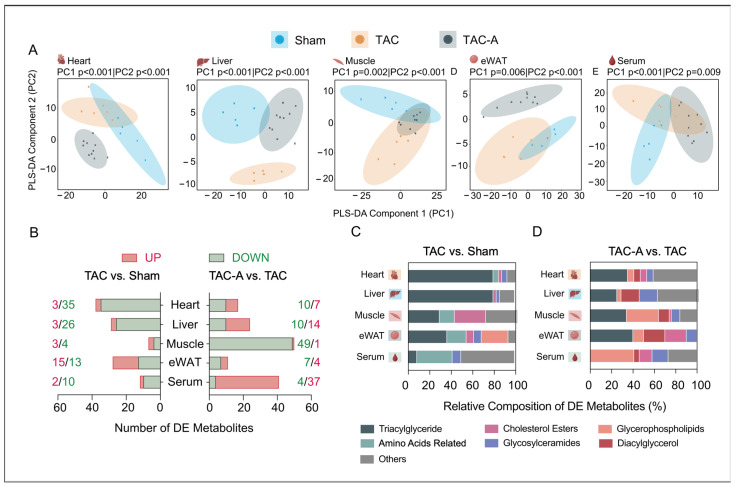
Tissue-specific metabolomic remodeling in pressure overload-induced cardiac dysfunction and its modulation by adiponectin receptor agonist ALY688. (**A**) Partial least squares discriminant analysis (PLS-DA) score plots illustrating separation of metabolite profiles across three experimental groups (Sham, TAC, TAC-A) in heart, liver, muscle, eWAT, and serum; (**B**) number of differentially expressed (DE) metabolites (upregulated: red; downregulated: green) across tissues in comparisons of TAC vs. Sham (left) and TAC-A vs. TAC (right)—DE metabolites were defined by unadjusted *p* < 0.05 and fold change > 1.2 or < 0.83; (**C**,**D**) relative composition of DE metabolites categorized per subclasses across tissues for TAC vs. Sham (**C**) and TAC-A vs. TAC (**D**).

Targeted metabolomics profiling across five tissues revealed distinct TAC-induced metabolic disruptions and partial restoration with ALY688 (Supplementary Table S1). Multivariate PLS-DA demonstrated clear separation among the Sham (*n* = 5), TAC (*n* = 5), and TAC-A (*n* = 10) groups in all tissues, indicating pronounced TAC-induced metabolic remodeling and partial restoration with ALY688 treatment (Figure 1A). TAC samples consistently deviated from Sham along principal component one, demonstrating a strong pressure overload-driven metabolic shift. In contrast, TAC-A samples partly overlapped with the Sham cluster, reflecting partial normalization of metabolic phenotypes following ALY688 administration.

To identify the metabolic features contributing to these group separations, a differential metabolite analysis was performed and summarized in a stacked bar plot (Figure 1B–D). TAC induced marked alterations in triglycerides (TGs), with more than 60 species significantly dysregulated, predominantly within the heart, liver, and eWAT. Additionally, 12 amino acid-related metabolites and over five hexosylceramides (HexCer) were differentially regulated across multiple tissues, highlighting broad tissue-specific metabolic reprogramming. ALY688 mitigated a substantial proportion of these TAC-driven alterations, partially restoring the dysregulated ceramide (Cer), HexCer, and cholesterol ester (CE) species and shifting TG and phosphatidylcholine (PC) remodeling patterns toward the basal state (Figure 1D). These findings indicate that ALY688 not only attenuates TAC-induced lipid accumulation but also rebalances tissue-specific lipid composition and systemic metabolic signaling.

Differentially expressed (DE) metabolites associated with TAC and ALY688 intervention were identified through pairwise comparisons (TAC vs. Sham and TAC-A vs. TAC) across all tissue types, using the significance threshold of *p* < 0.05 and fold change < 0.83 (downregulated) or > 1.2 (upregulated) (Table S1). The number of DE metabolites per tissue and comparison are summarized in the stacked bar plot (Figure 1D). Pressure overload predominantly affected TG, PC, and amino acid-related metabolites, while ALY688 altered patterns of DG, HexCer, and CE. These patterns highlight differential vulnerability and metabolic responsiveness of tissues to pressure overload and adiponectin receptor activation.

### 3.2. Differentially Expressed Metabolites Reveal Tissue-Specific Alterations in TAC

**Figure 2:**
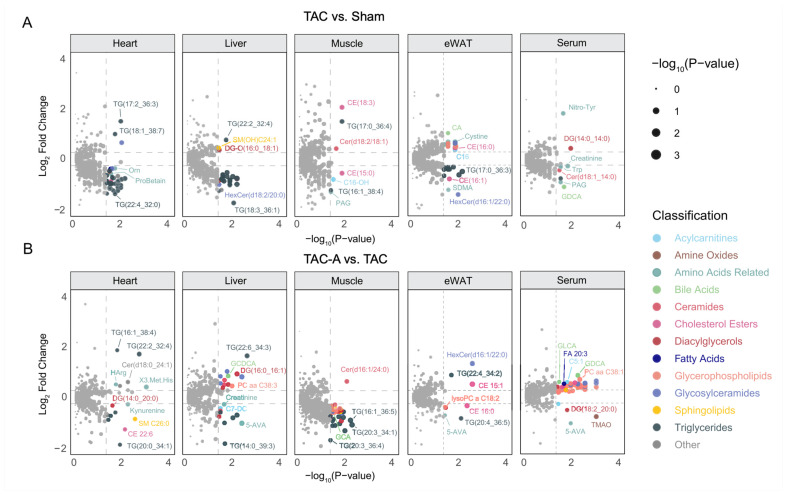
Differential metabolite profiles reveal tissue-specific metabolic perturbations induced by TAC and partial restoration with ALY688. (**A**,**B**) Volcano plots showing DE metabolites in TAC vs. Sham (**A**) or TAC-A vs. TAC (**B**) across heart, liver, muscle, eWAT, and serum. Each point represents an annotated metabolite, displayed as log_2_ FC vs. −log_10_(*p*-value). Metabolites are color-coded by subclass, highlighting tissue-specific metabolic disturbances; point size corresponds to −log_10_(*p*-value), emphasizing metabolites with greater statistical significance.

To further characterize tissue-specific metabolic disruptions induced by pressure overload and the restorative effects of ALY688, color-coded volcano plots were employed to visualize both the magnitude (log_2_ fold change) and significance (−log_10_ *p*-value) of metabolite alterations at the feature level across heart, liver, skeletal muscle, eWAT, and serum (Figure 2A,B). TAC vs. Sham comparisons revealed pronounced dysregulation of multiple lipotoxic and energy-related metabolites across all tissues (Figure 2A). In the heart, TAC markedly reduced ProBetaine. Triglycerides (TGs) exhibited heterogeneous responses: while a limited number of TG species, including TG(17:2_36:3), TG(18:1_38:7), and TG(17:0_36:4), were elevated, the majority of TGs were significantly downregulated in heart, liver, and eWAT. This widespread reduction in TG abundance suggests enhanced hydrolysis and depletion of TG stores, likely reflecting increased mobilization of fatty acids to meet elevated energetic demands in pressure-overloaded tissues.

Concurrently, liver, skeletal muscle, and eWAT exhibited elevated levels of several ceramide (Cer) and cholesterol ester (CE) species, including Cer(d18:2/18:1), CE(18:3), and CE(16:0), indicating pressure overload-induced lipid remodeling and accumulation of lipotoxic lipid species.

Across tissues, several gut-derived metabolites also showed significant alterations in the pairwise comparisons. Glycodeoxycholic acid (GDCA) was markedly reduced in TAC serum, indicating suppression of bile acid-associated metabolic signaling under pressure overload. ALY688 enabled significant restoration of GDCA in serum upon TAC. While TAC did not significantly alter trimethylamine N-oxide (TMAO) or 5-aminovaleric acid (5-AVA) levels, ALY688 treatment resulted in a marked reduction across several tissues, suggesting that modulation of gut-derived metabolites is specifically associated with ALY688-mediated metabolic restoration.

### 3.3. ALY688 Partially Restores TAC-Induced Metabolic Dysregulation Across Tissues

**Figure 3:**
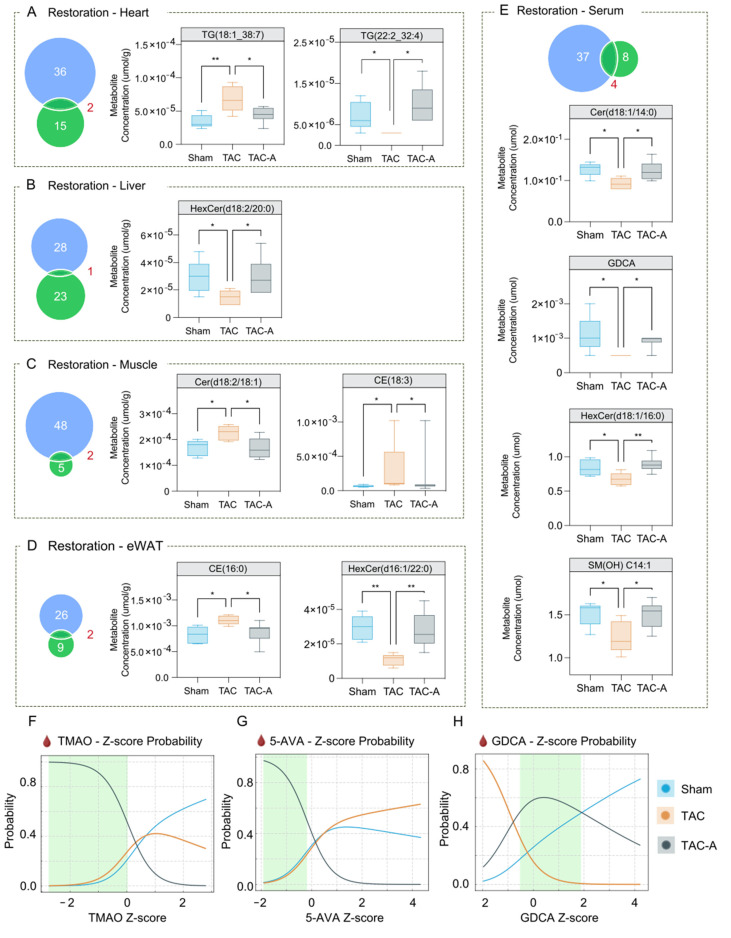
ALY688 restores TAC-induced metabolic alterations across tissues and normalizes circulating metabolite signatures associated with cardiac dysfunction. (**A**–**E**) Venn diagrams (left panels) show the number of metabolites altered by TAC (vs. Sham; blue) and those altered by ALY688 treatment (vs. TAC; green) across heart (**A**), liver (**B**), muscle (**C**), eWAT (**D**), and serum (**E**). The overlapping region represents metabolites meeting significance criteria in both comparisons, identifying metabolic features jointly associated with TAC and ALY688 treatment. Boxplots (right panels) display representative metabolites that were significantly dysregulated by TAC and subsequently reversed toward Sham-like levels with ALY688. Data are presented as metabolite concentrations (µmol/g for tissue or µmol for serum), showing recovery in the TAC-A group compared to TAC. (**F**–**H**) Multinomial logistic regression-based probability curves demonstrating group classification probability based on Z-scores of circulating metabolites TMAO (**F**), 5-AVA (**G**), and GDCA (**H**); shifts in probability profiles indicate treatment-associated transitions from a TAC-dominant metabolic signature toward a Sham-like state. Green-shaded regions denote relative ranges that preferentially distinguish TAC-A from both TAC and Sham groups. Statistical comparisons were performed using one-way ANOVA with Tukey’s post hoc test; * *p* < 0.05, ** *p* < 0.01.

Venn diagrams revealed substantial overlap between TAC-responsive metabolites and those altered by ALY688 in TAC-A, indicating that the peptide mitigates a meaningful portion of the TAC-induced metabolic disruption (Figure 3A–E). Notably, nearly all overlapping metabolites exhibited changes in TAC-A that were directionally opposite to those observed in TAC, supporting a restorative rather than compensatory metabolic effect. Bar graph comparisons further demonstrated that ALY688 reversed many TAC-induced alternations in Cer, HexCer, and CE across heart, liver, skeletal muscle, and eWAT. Across all four tissues, ALY688 consistently shifted TAC-altered lipid species toward Sham levels, indicating broad metabolic normalization.

To evaluate the discriminatory potential of key gut-derived metabolites, multinomial logistic regression (MLR) analysis was performed using serum levels of TMAO, 5-AVA, and GDCA (Figure 3F–H). Normalized probability curves illustrated a relative trend in which the TAC-A group (grey) was most distinguishable from both Sham (blue) and TAC (orange). Green-shaded regions in each curve represent metabolite intervals with the greatest discriminative value for identifying ALY688-treated TAC-A mice. These findings suggest that ALY688 exerts coordinated multi-organ effects, normalizing lipotoxic and gut-derived metabolic abnormalities associated with pressure overload.

### 3.4. Pathway-Level Analysis Identifies Key Metabolite Pathways

**Figure 4:**
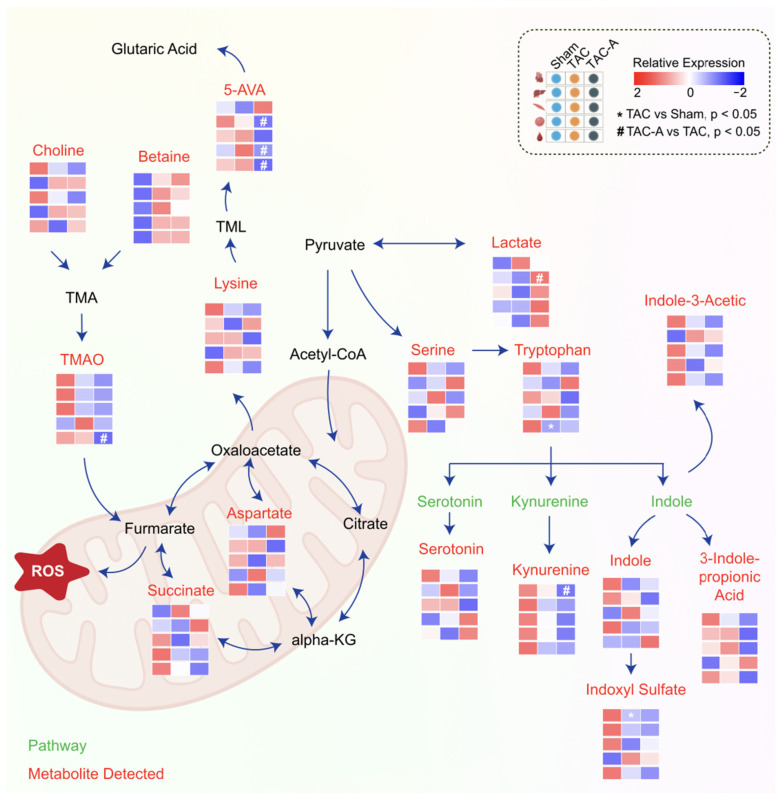
Overview of altered metabolic pathway by ALY688 in TAC-induced HFrEF. Schematic overview of altered metabolic pathways involving amino acid catabolism, TCA cycle intermediates, and gut microbiota-derived metabolites. Heatmaps show relative metabolite abundance across Sham, TAC, and TAC-A groups (red = high, blue = low). TAC induced elevation of key metabolites associated with mitochondrial stress and inflammation, including TMAO, 5-AVA, kynurenine, and succinate, alongside disruption of serine, tryptophan, and aspartate metabolism. ALY688 treatment partially restored these dysregulated metabolites toward Sham-like levels, suggesting improved mitochondrial metabolic balance and reduced gut–heart metabolic stress.

A schematic illustration of the metabolic network was created based on the common metabolic pathways to delineate the metabolic rewiring across all tissues in TAC-induced pressure overload, with or without ALY688 (Figure 4). The network encompasses metabolic pathways enriched with DE metabolites identified in this study. Specifically, the interplay between TMAO, 5-AVA, the TCA cycle, and tryptophan metabolism emerged as key pathways modulated by ALY688, suggesting the potential involvement of a gut–liver–heart metabolic axis.

## 4. Discussion

Numerous studies have investigated therapeutic strategies for HFrEF, including the cardioprotective potential of the adiponectin receptor agonist ALY688, in preclinical models of HFrEF [11,19,20]. TAC induces systemic metabolic remodeling, contributing to insulin resistance, hypertrophy, inflammation, and fibrosis. Adiponectin and its agonists have been shown to mitigate these factors, thereby protecting cardiac function [21]. Studies have established that adenoviral-delivered or recombinant adiponectin can regulate these contributory factors and thus confer cardioprotective effects [11,22,23]. In this study, we used targeted LC–MS/MS metabolomics across multiple tissues (heart, liver, skeletal muscle, eWAT, serum) to comprehensively examine systemic metabolic remodeling induced by TAC and to assess how treatment with the adiponectin receptor agonist ALY688 influences these changes.

Our findings suggest that TAC induces profound metabolic alterations in lipid species, especially Cer, HexCer, and CE, across multiple organs and that ALY688 treatment can partially restore many of these aberrant metabolite profiles. Moreover, we identify the involvement of gut-derived metabolites (e.g., TMAO, 5-AVA, GDCA), suggesting that the gut–liver–heart metabolic axis is engaged in TAC and responds to ALY688 treatment. These effects were reflected not only in metabolite abundance but also in shifts in the overall metabolic profiles, as shown by the partial separation between Sham and TAC-A across all tissues in the PLS-DA plots.

Our results build on the growing recognition that pathological metabolic remodeling may be one of the central drivers of HF progression, not just at the level of the heart but systemically [24,25]. TAC clearly reprogrammed the metabolome in a coordinated, tissue-specific manner, where the heart and liver showed the greatest number of differentially expressed metabolites, while skeletal muscle, eWAT, and serum also manifested significant changes. In line with this, TGs and PCs exhibited marked tissue-dependent remodeling, with multiple TG species reduced in the heart and several PCs altered across liver, muscle, eWAT, and serum. This aligns with prior work demonstrating that chronic cardiac stress alters systemic metabolism and insulin sensitivity, promoting lipotoxicity and maladaptive lipid accumulation [25,26].

Indeed, previous studies in HF models have reported upregulation of lipid-metabolizing enzymes in the failing heart and lipid overload as a contributor to disease progression [27,28]. One compelling feature in the data was the accumulation of Cer species in TAC hearts relative to Sham. Elevated Cers are known to contribute to cardiometabolic pathophysiology [29,30,31]. These results are consistent with mechanistic studies of adiponectin receptor agonists; for example, AdipoRon (another adiponectin receptor agonist) has been shown to enhance ceramidase activity and reduce Cer accumulation in diabetic cardiomyopathy, improving insulin resistance, reducing fibrosis and inflammation, and promoting cardiac function [27,32]. However, long-term AdipoRon treatment has also been associated with dose-dependent toxicities and paradoxical effects on insulin sensitivity, highlighting limitations of its therapeutic potential [33,34].

In the current model, ALY688 treatment restored several TAC-induced lipotoxic lipid species across peripheral tissues, including Cer(d18:2/18:1), cholesteryl ester CE(18:3), and multiple hexosylceramide species such as HexCer(d18:2/20:0) and HexCer(d18:1/16:0). The Venn diagram analysis further supported this finding, showing that nearly all overlapping TAC-altered metabolites exhibited a restorative direction of change under ALY688 treatment. Collectively, these findings indicate that ALY688 reverses a ceramide-dominant sphingolipid imbalance induced by pressure overload, consistent with normalization of systemic lipid homeostasis. Hence, the observation is in line with ALY688’s known cardioprotective effects in preclinical HFrEF models, where it attenuates fibrosis, hypertrophy, and inflammation [11]. Thus, our metabolomic findings provide mechanistic support for ALY688’s beneficial effects. These restorative shifts were also apparent in TG and PC remodeling, particularly the increase in several TG species in TAC-A hearts compared to TAC and normalization of PC species across tissues.

Additionally, the data showed the involvement of gut-derived metabolites, which have been connected to cardiovascular disease outcomes [35,36]. In TAC, we observe elevated TMAO and 5-AVA and altered GDCA profiles, consistent with dysregulation of gut–liver–heart metabolic communication [18,37,38]. In serum, GDCA showed a clear pattern, with TAC decreasing GDCA and ALY688 restoring its abundance toward Sham levels. ALY688’s restoration effect indicates that adiponectin receptor activation may influence not just intracellular lipid metabolism but also systemic metabolic axes that involve the gut microbiome and bile acid metabolism. Although our findings demonstrate consistent shifts in GDCA, TMAO, and 5-AVA, we cannot directly infer that these metabolite changes causally mediate cardioprotection. Prior literature suggests potential mechanisms, including modulation of bile acid receptor signaling, endothelial activation, inflammatory cytokine regulation, and microbial amino acid metabolism, but these pathways were not tested experimentally in the present study [17,18,37]. Z-score probability curves further demonstrated that TAC-A displayed distinct distributional patterns relative to TAC and Sham, highlighting metabolite ranges that most effectively discriminated against the ALY688-treated group. The normalization of TMAO and 5-AVA suggests potential improvements in microbial flux, choline metabolism, or hepatic handling of gut-derived metabolites [17,32].

These systemic effects point to a model in which ALY688 promotes metabolic homeostasis both inside the heart, through reducing lipotoxic intermediates, and in circulating compartments, through restoring gut–metabolism crosstalk. The consistent multi-tissue shifts observed in the PLS-DA, heat maps, volcano plots, and probability curves indicate that ALY688 produces coordinated changes rather than isolated tissue effects. Such actions may underline its observed anti-inflammatory and anti-fibrotic benefits by reducing ceramide stress, improving lipid handling, and modulating gut-derived metabolites. Furthermore, our data may help explain how ALY688 confers its cardioprotective effects in vivo*,* which was observed in preliminary data where ALY688 was shown to reduce markers of fibrosis, hypertrophy, and inflammation [11]. The participation of gut-derived metabolites underscores that therapies targeting adiponectin signaling may repair not only heart-intrinsic pathology but also broader systemic metabolic derangements. Therefore, the results of this study align with a growing body of evidence showing that HF is not just a hemodynamic disease but also a metabolic and inflammatory disorder that involves crosstalk among tissues [20,27]. Taken together, the coordinated, multi-tissue metabolic restoration observed with ALY688 administration provides a clear framework for considering its translational value. As circulating metabolites are increasingly recognized as biomarkers of disease severity and therapeutic response in HF, the metabolic signatures identified here may inform patient stratification and prognosis. In this context, adiponectin receptor agonism represents a promising systemic therapeutic strategy that extends beyond cardiac-specific mechanisms to address broader metabolic dysregulation associated with HFrEF.

While these results provide important mechanistic insight into systemic metabolic remodeling in pressure overload-induced HFrEF and its modulation by ALY688, several limitations should be acknowledged. First, the current study was conducted exclusively in male mice. Given the well-established sex differences in cardiometabolic regulation, adiponectin signaling, and HF progression, these findings may not be directly generalizable to females [39,40]. Future studies incorporating both sexes in parallel will be essential to determine whether the observed metabolic signatures and therapeutic responses to ALY688 exhibit sex-specific effects. Second, although the targeted metabolomics platform enabled absolute quantification of a defined panel of annotated metabolites with high analytical confidence, its targeted nature inherently limited metabolic discovery. Novel, unexpected, or low-abundance metabolites and pathways may not have been captured. Integration of complementary untargeted metabolomics approaches could provide broader coverage and reveal additional metabolic networks relevant to disease progression and therapeutic response. Third, the experimental design included Sham, TAC, and TAC with ALY688 groups but did not include a Sham with ALY688 group. Consequently, it is not possible to definitively distinguish whether ALY688 primarily normalizes pressure overload-induced metabolic disturbances or exerts independent pharmacological effects in the absence of pathological stress. Inclusion of a Sham + ALY688 group in future studies will be important to clarify treatment specificity and to differentiate normalization from baseline metabolic modulation, if any. Additionally, the exploratory nature of the metabolomic analysis involved a larger number of statistical comparisons. Although a combined criterion of nominal *p*-value < 0.05 and FC threshold was applied to improve robustness, the absence of formal multiple testing correction (e.g., false discovery rate) increases the risk of false-positive findings. The larger treatment group was intentionally used to increase precision in estimating the magnitude and variability of ALY688’s effect across the multi-tissue metabolomics endpoints and to improve robustness of treatment–response inference while maintaining appropriate control and disease comparator groups. Accordingly, the identified differentially abundant metabolites should be interpreted as hypothesis-generating and will require follow-up validation in independent and larger-scale cohorts. Finally, although gut-derived metabolites emerged as key discriminators between experimental groups, the study design did not include complementary microbiome profiling. The lack of 16S rRNA gene sequencing or metagenomic analysis precludes direct assessment of whether TAC or ALY688 altered microbial composition or functional capacity. Integrating microbiome sequencing with metabolomics in future studies will be critical to determine whether the observed metabolite changes reflect host-driven metabolic regulation, microbiota-dependent effects, or interactions between the two.

## 5. Conclusions

In conclusion, targeted multi-organ metabolomics reveals that pressure overload induces widespread metabolic remodeling, particularly in lipids (CE, HexCer, Cer) and gut-derived molecules (TMAO, 5-AVA, GDCA). Treatment with the adiponectin receptor agonist ALY688 partially normalizes these maladaptive metabolic signatures in multiple tissues, restoring aspects of metabolic homeostasis. Therefore, this data provides important mechanistic insight into how ALY688 may exert cardioprotective effects and supports further preclinical and translational exploration of adiponectin receptor agonism as a therapeutic strategy in HFrEF.

## Data Availability

The data presented in this study are available upon reasonable request from the authors.

## Supplementary Materials

The following supporting information can be downloaded at: https://www.mdpi.com/article/10.3390/metabo16010038/s1, Table S1: List of differentially expressed metabolites associated with TAC and ALY688 intervention.
